# The influence of hormonal changes during pregnancy on bone metabolism - a mini review

**DOI:** 10.3389/fendo.2026.1819544

**Published:** 2026-06-18

**Authors:** Anna Brona, Barbara Stachowska, Marek Bolanowski, Jowita Halupczok-Żyła

**Affiliations:** 1 Department and Clinic of Endocrinology and Internal Medicine, Wrocław Medical University, Wrocław, Poland; 2 Department of Clinical and Experimental Pathology, Wrocław Medical University, Wrocław, Poland

**Keywords:** ACTH, cortisol, estrogen, growth hormone, IGF-1, prolactin, PTHrP, TSH

## Abstract

During pregnancy, the maternal endocrine system undergoes a profound hormonal transformation to support fetal development. Although these changes are essential, they significantly affect maternal bone metabolism and may result in a rare but serious condition known as Pregnancy and Lactation-Associated Osteoporosis (PLO). Despite the change in maternal hormone production, there is a new player – a placenta, which makes a substantial contribution to the bone remodeling process. Most publications address the influence of parathyroid hormone, prolactin, and growth hormone. However, data on the interactions between other hormones and skeletal system cells remain limited. Our mini-review focuses on the mechanism by which hormones influence the activity of osteoclasts and osteoblasts.

## Introduction

Pregnancy is a state of significantly changed physiology. Hormonal changes are necessary for fetal growth. Some changes are supposed to contribute to bone fragility; however, data on the actions of hormones on bone are limited (1). In some women, pregnancy and lactation lead to osteoporosis. This is an important clinical and social issue, though we have decided to summarize current data on how hormonal changes contribute to bone fragility. Pregnancy and lactation-associated osteoporosis (PLO) is a rare condition diagnosed in premenopausal women in the third trimester of pregnancy or during lactation, especially in the early phase of lactation. The most recent studies on the epidemiology of PLO report rates of 4.5/10,000 and 4.6/10,000, while previous studies suggested rates of 4–8 per million pregnancies (2–4). PLO is thought to be associated with addressing the increased demand for calcium for the fetus in pregnancy and for milk during lactation. In general, changes observed in pregnancy and lactation do not lead to osteoporosis and fractures; however, in some women, osteoporosis with fractures occurs. It is suggested that concomitant conditions and diseases, as well as genetic predisposition existing before pregnancy, contribute to PLO. However, most women do not have any predisposing risk factors (1). Recently, a few important reviews have been published (1, 5, 6). They discuss mainly calcium homeostasis in pregnancy and lactation linked to parathormone (PTH) and parathyroid hormone-related protein (PTHrP) actions, with brief information on other hormones involved in bone metabolism. Therefore, we would like to present data on hormonal influence on bone other than PTH that may contribute to PLO or protect against PLO.

## PTH, PTHrP and calcitonin

PTH decreases to the low-normal range during the first trimester and, at the end of pregnancy, may rise to the mid-normal range in women consuming calcium-replete diets (7). PTH exerts effects on osteoblasts through PTH1 receptors. There are no PTH receptors on osteoclasts. Within osteoblasts, PTH promotes the synthesis of receptor activator of nuclear factor κB ligand (RANKL) and suppresses the synthesis of osteoprotegerin (OPG). PTH regulates both bone formation and resorption, depending on the mode of exposure (continuous or intermittent), favoring osteoclast differentiation and activity or osteoblast proliferation and differentiation. The placenta and breasts are the most essential sources of PTHrP. It is supposed that PTHrP may enhance the renal 1α-hydroxylase, followed by the rise of calcitriol, though its actions seem less effective than PTH (8). PTHrP levels rise progressively in maternal circulation and reach their peak during the third trimester (7, 9). PTHrP exerts some of the actions of PTH, e.g. bone resorption. It also enhances calcitriol production, though it is less effective than PTH due to structural differences. However, homology between PTH and PTHrP is enough to bind to the same PTH-1 receptor (PTH1R) (10). An *in vitro* study also revealed that a carboxyl-terminal form of PTHrP suppresses osteoclastic bone resorption and may help protect against excessive bone loss during pregnancy (11). Serum calcitonin concentrations rise during pregnancy and appear to originate not only from the maternal thyroid but also from the breast and placenta (12, 13). It has been demonstrated that in pregnant women who underwent thyroidectomy before pregnancy, calcitonin concentrations increased from undetectable to normal (12). PTHrP actions primarily increase calcitriol production and enhance renal calcium absorption. PTH actions are similar, however its concentration decreases in pregnancy. The actions of PTH and PTHrP may be bidirectional, i.e. promote both osteoblastogenesis and osteoclastogenesis. Thus, PTHrP may be distinguished as mayor player for PLO as continuous release rises concern about the enhanced formation of osteoclast. In contrast, one form of PTHrP is considered to inhibit osteoclast production. Calcitonin exerts its effects through a G protein–coupled calcitonin receptor, which activates the cyclic adenosine monophosphate (cAMP) and phospholipase C/inositol 1,4,5-trisphosphate (PLC/IP_3_) pathways (14). In the short term, it modifies osteoclasts’ sensitivity to circulating calcium (15). Calcitonin also promotes osteoclasts’ contraction, contributing to their impaired motility. Finally, it suppresses osteoclast-driven bone resorption. It also suppresses carbonic anhydrase II and disrupts the acidic conditions required for osteoclast actions. Furthermore, calcitonin impairs the differentiation of osteoclast precursors (14). The role of calcitonin in calcium homeostasis in pregnancy remains uncertain. Although it has been suggested that calcitonin may help to protect the maternal skeleton from excessive resorption, this has not been confirmed in clinical studies. It is also unknown if calcitonin contributes to pregnancy and lactation-associated osteoporosis. Interestingly, calcitonin-deficient mice exhibit normal calcium and bone metabolism during pregnancy (16, 17). Further investigation is needed to determine whether it could be recognized as a protective factor for bone health in pregnancy.

## Calcitriol, FGF23

During pregnancy, maternal adaptation to increase calcium absorption is most efficiently mediated by elevated 1,25-dihydroxyvitamin-D (1,25(OH)_2_D, calcitriol) levels (7). Data suggest that free calcitriol levels are higher in the third trimester, while total calcitriol levels increase two- to five-fold early in pregnancy and remain elevated until delivery (18). Other authors have suggested that free calcitriol is increased in all three trimesters (19, 20). Maternal renal 1α-hydroxylase plays the most important role in calcitriol production (7, 21). CYP27B1 (the gene that encodes 1α-hydroxylase) is crucial for converting 25-hydroxyvitamin D into active 1,25-dihydroxyvitamin D. Pregnancy induces a 30-fold higher expression of CYP27B1 in the maternal kidneys compared to the placenta (22). PTH is normally the dominant regulator of CYP27B1, but the marked pregnancy-related increase in calcitriol occurs while PTH is suppressed to low levels (23). Data from animal models indicate that 1α-hydroxylase-mediated calcitriol synthesis is regulated by other factors, including PTH-related protein (PTHrP), estradiol, PRL, and placental lactogen (7). CYP24A1 (the gene encoding 24-hydroxylase) balances this by accelerating the inactivation of this excess vitamin D (7). Fibroblast growth factor 23 (FGF23) is a hormone that is mainly secreted by osteocytes and osteoblasts in bone (24). A small longitudinal study of 12 women revealed that intact FGF23 doubled in the third trimester compared to values in the first and second trimesters (25). However, data from a larger longitudinal study of 81 women revealed no difference in intact FGF23 values at 36 weeks of gestation versus 3–6 months post-weaning (26). FGF23 regulates phosphate levels during pregnancy by reducing renal phosphate reabsorption and suppressing 1,25(OH)_2_ vitamin D production (27).

*In vitro* study has reported that the overexpression of FGF23 reduces the proliferative capacity of human bone marrow mesenchymal stem cells and the expression of key osteogenic differentiation genes, such as RUNX2, OCN, and OSX (28).

FGF23 reduces serum 1,25(OH)_2_D levels by downregulating CYP27B1 expression, which limits 1α-hydroxylase protein levels. Simultaneously, it upregulates CYP24A1, leading to increased vitamin D catabolism via 24-hydroxylase (29). However, markedly increased FGF23 leads to hypophosphatemia and low 1,25(OH)_2_D levels, which may ultimately result in impaired mineralization of the bone matrix. Maternal iron deficiency is associated with the increased expression of FGF23. Since iron-deficiency anemia is a common condition in pregnancy, an increase in FGF23 is likely to be found in many pregnant women (30). Currently, there are no data confirming that FGF23 causes PLO. However, given its mechanism of action, it is likely to contribute to the pathogenesis of the condition.

## Prolactin/lactogen

**Figure 1:**
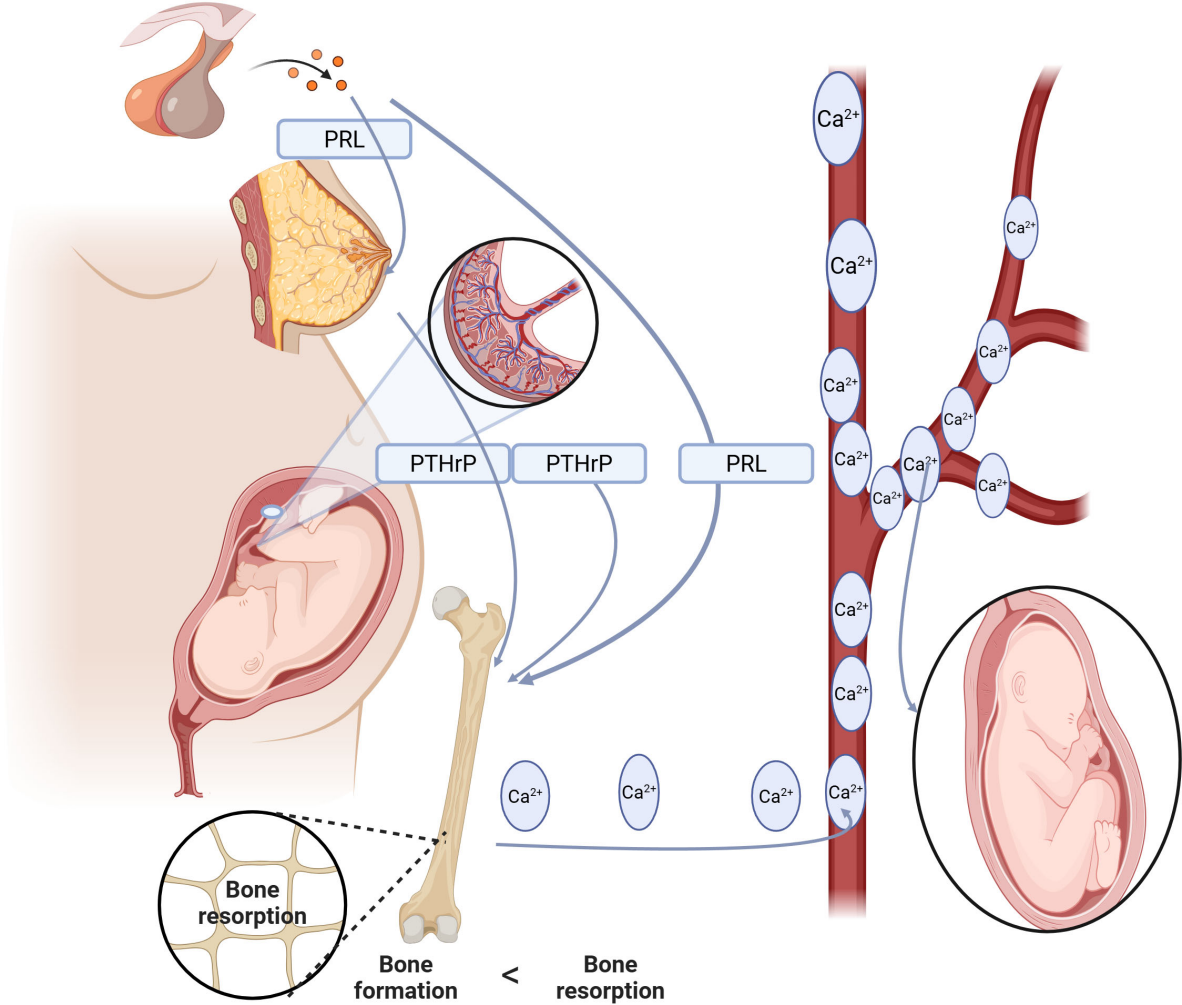
PTHrP and PRL actions involved in bone metabolism during pregnancy. The placenta and breasts are the most essential sources of PTHrP. PRL stimulates PTHrP synthesis and release from the breasts. PTHrP, parathyroid hormone-related protein; PRL, prolactin. Figure was created with BioRender.com.

**Figure 2:**
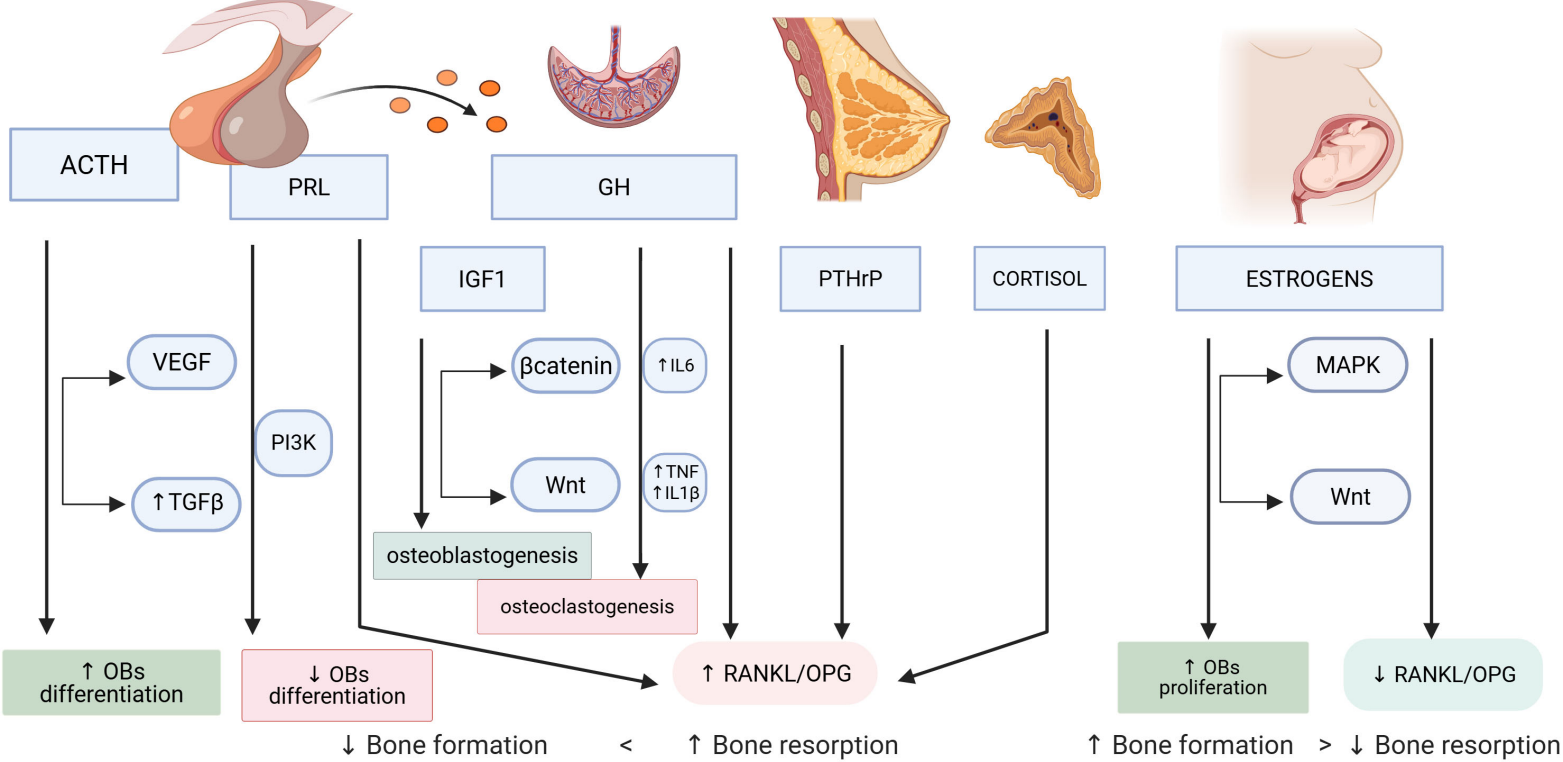
Hormonal and bone changes observed in pregnancy. The molecular mechanisms underlying hormone actions are complex, as hormones promote both osteoclastogenesis and osteoblastogenesis. Osteoblast differentiation is driven by estrogens, IGF-1 and ACTH. Positive effects on osteoclasts are mediated by PRL, GH, cortisol and PTHrP. Hormones act via several distinct mechanisms, i.e. the RANKL-OPG system, cytokines, Wnt signaling and growth factors. TGF-β is enhanced by ACTH. At the same time it is downregulated by cortisol. The positive actions of estrogens are mediated by Wnt-signaling. Cortisol exerts opposing effects on these pathways. IGF-1, insulin-like growth factor-1; ACTH, adrenocorticotropic hormone; PRL, prolactin; GH, growthhormone; PTHrP, parathyroid hormone- related protein; RANKL, receptor activator for nuclear factor κ B ligand; OPG, osteoprotegerin; Wnt, Wingless-related integration site; TGF-β, transforming growth factor β; VEGF, vascular endothelial growth factor; OBs, osteoblasts; PI3K, phosphoinositide 3-kinase; IL-6, interleukin 6; TNF, tumor necrosis factor; IL-1β, interleukin 1β; MAPK, mitogen-activated protein kinase. Figure was created with BioRender.com.

**Figure 3:**
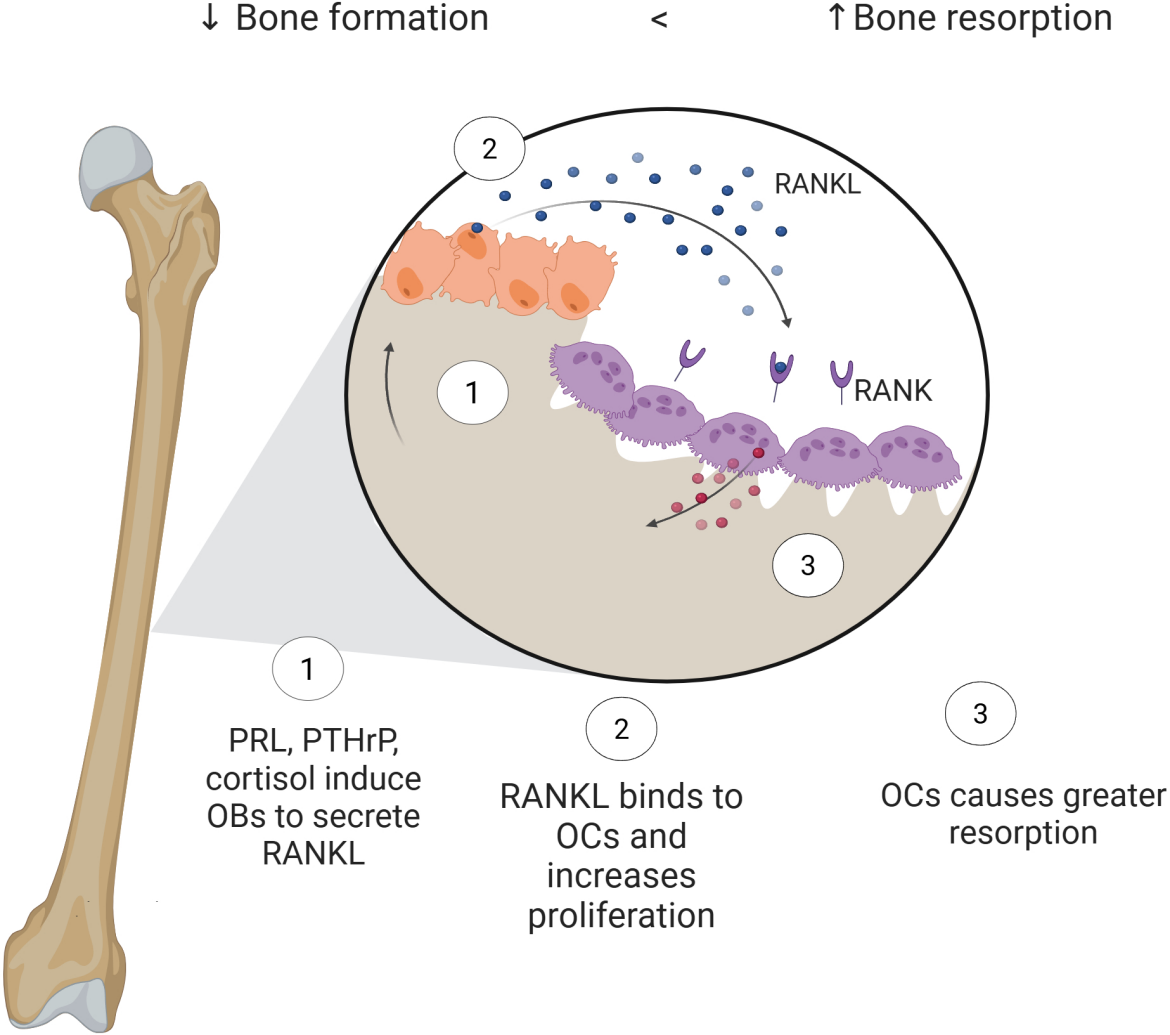
The RANKL-OPG system. Osteoblasts express soluble and membrane-bound RANKL which binds to RANK in the membrane of osteoclast precursors. RANK signaling activates osteoclasts differentation. RANKL synthesis is upregulated by PTHrP, cortisol and PRL. The adverse effect on RANKL is mediated by estrogens. Most hormones which modify RANKL concentration regulates also OPG concentrations, i.e. TSH and estrogens increase OPG levels, whereas PTHrP, cortisol and PRL reduce them. RANKL, receptor activator for nuclear factor κ B ligand; OPG, osteoprotegerin; RANK, receptor activator for nuclear factor κ B; PTHrP, parathyroid hormone- related protein: PRL, prolactin; TSH, thyroid-stimulating hormone; OBs, osteoblasts; OCs, osteoclasts. Figure was created with BioRender.com.

**Figure 4:**
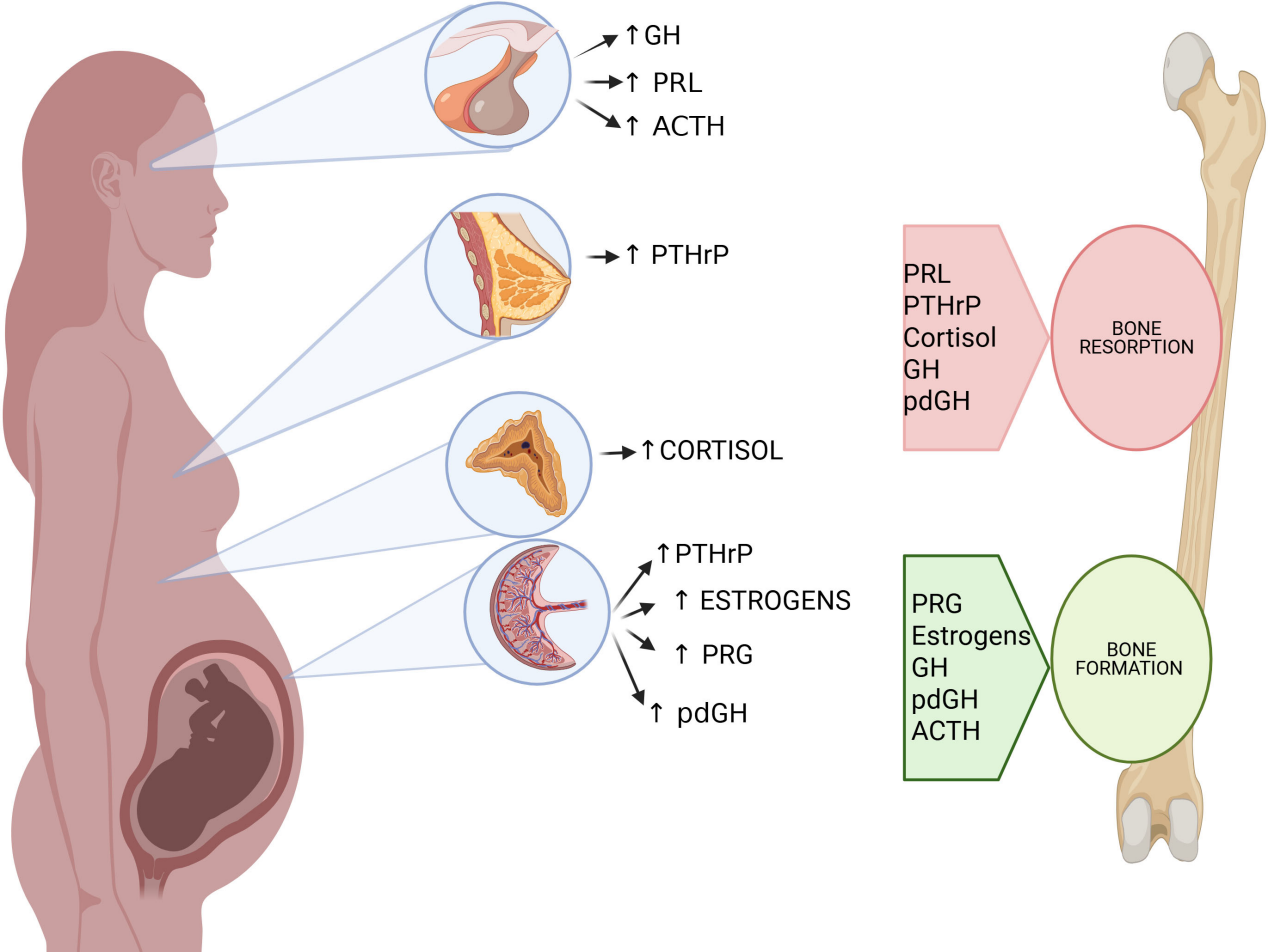
Changes in hormone concentrations during pregnancy – an increase in PTHrP, PRL, cortisol, ACTH, GH, progesterone and estrogen. The placenta and breasts are the most essential sources of PTHrP. Increased PRL concentrations contribute to bone resorption in pregnancy. PRL actions are multiple, both direct through PRL receptors on osteoblasts and indirect on osteoclasts due to regulation of RANKL, OPG and osteoclastogenic modulations. In pregnancy, two variants of GH are expressed: pituitary-derived GH and pdGH. GH and IGf-1 promote osteoblastogenesis but also osteoclastogenesis. ACTH levels increase due to placental production of ACTH and CRH. These changes also lead to increased cortisol levels, ACTH exerts a positive effect on osteoblast both through receptors and network of regulatory proteins but cortisol levels resulting in the opposite effect. Estrogen and progesterone concentrations in pregnancy may be considered highly favorable for protecting against bone loss. PTHrP, parathyroid hormone- related protein; PRL, prolactin; ACTH, adrenocorticotropic hormone; GH, growth hormone; RANKL, receptor activator for nuclear factor κ B ligand; OPG, osteoprotegerin; pdGH, placenta-derived growth hormone; IGF-1, insulin-like growth factor-1; CRH, corticotropinreleasing hormone; PRG, progesterone. Figure was created with BioRender.com.

Human placenta lactogen (hPL) and PRL (PRL) increase during pregnancy. They affect skeletal metabolism during pregnancy (23). They activate PRL receptors on osteoblasts (31). Additionally, PRL stimulates PTHrP synthesis and release from the breasts (31, 32). PRL levels increase steadily throughout pregnancy due to hyperplasia of pituitary lactotrophs stimulated by estrogen (33). PTHrP and PRL actions involved in bone metabolism during pregnancy are outlined in **Figure 1**. hPL concentration increases during pregnancy, with the highest levels observed in the third trimester. It is produced by syncytiotrophoblast cells (34). PRL receptors are present on osteoblasts (35). In a study of human pre-osteoblast cells, it was found that PRL markedly suppresses osteoblast proliferation and, at the same time, enhances bone formation markers, i.e. runt-related transcription factor 2 (RUNX2) and alkaline phosphatase (ALP) during the initial phases of osteoblast differentiation. However, PRL decreases these bone formation markers during the later phases of osteoblast differentiation (36). A decrease in osteoblast differentiation markers induced by PRL is mediated, at least in part, by the phosphoinositide 3-kinase (PI3K) signaling pathway (37, 38). Hormonal and bone changes observed in pregnancy are detailed below and outlined in **Figures 2**, **3** and **4**. In general, an excess of PRL is correlated with osteoporosis in hyperPRLemic conditions (39). In terms of PRL concentration pregnancy mimics hyperPRLemia in non-pregnant women. Increased PRL level in pregnancy is a physiological adaptation and it is not considered as pathological hyperPRLemia, despite its level overcomes PRL level in non-pregnant women (40). However, in mice without PRL receptor (Prlr -/-), lower BMD was demonstrated regardless of sex hormone concentrations (41). Mice lacking PRL receptors showed decreased bone formation. Osteoclasts do not express PRL receptors, and PRL exerts its action indirectly by elevating RANK ligand levels and decreasing OPG levels (35, 38). It also contributes to the upregulation of osteoclastogenic modulators, i.e. monocyte chemoattractant protein-1 (MCP-1), cyclooxygenase-2 Cox-2, tumor necrosis factor α (TNF-α), interleukin - 1 (IL-1), and ephrin-B1 (42). In another study in goldfish, it was demonstrated that high PRL concentrations were associated with increased activity of osteoclasts, while low PRL concentrations caused decreased activity (43).

Increased PRL concentrations contribute to bone resorption in humans during pregnancy and beyond. PRL has multiple actions, both direct through PRL receptors on osteoblasts and indirect on osteoclasts due to regulation of RANKL, OPG and osteoclastogenic modulators. It is regarded as a key factor in PLO.

One of the very important PRL actions is stimulation of PTHrP production and release. Placenta-derived GH supports and amplifies the effects of PRL by activating PRL receptors (34). These interactions of hormones additionally contribute to the final decrease in mineral bone density and may result in PLO.

## Growth hormone and IGF-1

In pregnancy, two variants of growth hormone (GH) are expressed: pituitary-derived GH and placenta-derived GH. This isoform can activate both the GH and PRL receptors (44). Pituitary GH secretion is pulsatile while placental GH release is steady (45). However, pituitary GH levels decline in pregnancy, and placental GH remains the dominant GH since about the 4th month of pregnancy. It is secreted continuously and increases until late pregnancy. In humans, most studies have demonstrated a significant increase (45-200%) in maternal insulin-like factor 1 (IGF-1) levels during the third trimester compared with non-pregnant women (46). hPL has GH activity. It binds to GH receptors with a considerably lower affinity than to PRL receptors (34). hPL does not exert such a strong effect on the secretion of IGF-1 as placental GH (47). IGF-1 augments osteoblast functional differentiation through direct signaling effects and indirectly by promoting β-catenin stabilization. β-catenin interferes with the WNT signaling pathway that promotes osteoblastogenesis (48). In osteoblast cultures, IGF-1 stimulates type I collagen gene expression and suppresses synthesis of matrix metalloproteinase-13, a key collagen-degrading protease (49). In genetically modified mice lacking IGF-1 receptor (IGF-1r) in osteoblasts, impaired bone formation and decreased trabecular bone volume have been observed (50). Another investigation in mice revealed that systemic IGF-1 supports cortical bone integrity, while locally synthesized skeletal IGF-1 is essential for maintaining trabecular bone integrity (51). Notably, human pre-osteoclasts express IGF-1 receptors, however the direct effects of IGF-1 on these cells require further investigation (52). IGF-1 promotes osteoclast formation and bone resorption *in vitro* (53). Importantly these effects occur indirectly, as IGF-1 acts on osteoblasts to increase the production of RANKL, the key driver of osteoclast maturation (**Figures 2**–**4**). Additionally, GH and IGF-1 stimulate IL-6 synthesis in human osteoblasts, and TNF α and IL-1β synthesis in peripheral blood mononuclear cells (53). TNF α supports osteoclastogenesis and bone resorption, leading to a negative balance in bone remodeling (53). Beyond these actions, GH and IGF-1 help maintain calcium balance through their ability to enhance renal vitamin D activation. In addition, GH stimulates tubular phosphate reabsorption (54). Investigations in mice without GH, the GH receptor (GHR), and IGF-1 indicated that GH and IGF-1 exert both independent and overlapping actions on the skeleton. Mice with combined GHR/IGF-1r deletion demonstrated more pronounced growth retardation than mice only deficient in GHR or IGF-1r (55). Furthermore, IGF-1 is essential for the anabolic actions of PTH on bone *in vivo*, as mice with IGF-1 inactivation do not demonstrate the expected bone-forming response to PTH (56).

Overall, GH and IGF-1 actions on osteoblasts and osteoclasts do not result in bone mass loss. They act directly and indirectly. Although they promote not only osteoblastogenesis but also osteoclastogenesis, it should be noted that both bone formation and resorption are required to maintain optimal bone quality. Their positive influence on bone health is also enhanced due to vitamin D activation in the kidney and the supporting effects of PTH on bone formation.

## FSH, estrogen and progesterone

### FSH

Follicle-stimulating hormone (FSH) levels fall significantly during pregnancy because the hypothalamic-pituitary axis is suppressed by high estrogen and progesterone production in the placenta. Estradiol levels rise dramatically during pregnancy because of placental estrogen synthesis and increased maternal adrenal precursor production. Progesterone levels rise similarly due to corpus luteum production in early pregnancy and placental production after the 10th week. FSH-receptors (FSHR) are expressed in osteoclasts and mesenchymal stem cells (57). FSH stimulates osteoclastogenesis and bone resorption and inhibits bone formation by FSHR, which belongs to the family of G-protein-coupled receptors (58–60). Studies in mice demonstrated that animals without FSH β and FSHR receptor had higher bone mass than wild-type animals (59). Female and male mice lacking FSH β had higher bone mass but narrower trabecular spacing (61). In another study, a humanized monoclonal anti-FSHβ antibody (MS-Hu6) decreased osteoclast formation (62). FSH upregulates RANK expression and supports osteoclasts differentiation, acting through TNF-α and IL-1β, and IL-6 (58, 63–65). It also enhances 3 established osteoclastogenic pathways by increasing the phosphorylation of Erk1/2, IκBα, and protein kinase B, thereby promoting osteoclast formation (59). FSH inhibits osteoblast differentiation through its receptor on mesenchymal stem cells. Anti-FSH antibodies stimulated bone formation, i.e. by promoting osteogenic genes (58, 62). An activating mutation of the FSHR was associated with a higher prevalence of osteoporosis. In postmenopausal AA rs6166 women, lower femoral neck bone mineral density (BMD) and higher serum concentration of ALP and C-terminal telopeptide of type 1 collagen (CTx) were observed (66). There is no data for pregnant women, but in pre-, peri-, and postmenopausal women, FSH concentrations and BMD were negatively correlated (67).

### Progesterone

Progesterone receptors (PRs) can be found in osteoblasts and osteoclasts (68, 69). Estrogen can upregulate progesterone (PR) expression in osteoblasts, raising the possibility that some effects usually attributed to estrogen on bone physiology may actually be mediated, at least in part, through enhanced progesterone signaling (68, 70). There is evidence that progesterone increases osteoblast production of transforming growth factor β 1–3 mRNA and bone-specific alkaline phosphatase, contributing to bone formation. Additionally, progesterone modulates metalloproteinase activity in human osteoblast-like cell cultures and may affect osteoblastogenesis or matrix remodeling (71). In human studies, it has been demonstrated that in premenopausal women, lower BMD correlates with lower progesterone levels (72).

### Estrogens

In osteoblasts, most of estrogen’s protective actions are mediated by estrogen receptor-alpha (ERα) (73). Numerous estrogen-responsive genes have been identified in osteoblasts (74). Estrogen promotes the expression of alkaline phosphatase and type I collagen. It also regulates the sensitivity of osteoblastic receptors to 1,25(OH)_2_D_3_ and PTH (75). In addition, estrogen has been shown to have anti-apoptotic effects on osteoblasts through acting on MAPK signaling pathways, transcription factors such as c-Jun and c-Fos, and Wnt signaling (75). Estrogens promote osteoblastogenesis by reducing intracellular reactive oxygen species (ROS) levels (76, 77). However, a principal action of estrogens is the decrease of the RANKL/OPG ratio. They suppress RANKL expression in osteoblasts and osteocytes and enhance the synthesis of the decoy receptor OPG. They also regulate the secretion of cytokines necessary for osteoclastogenesis (e.g., IL-1, IL-6, TNF-α, and M-CSF) (77–80) (**Figures 2**–**4**).

FSH, estrogen, and progesterone concentrations in pregnancy may be considered highly favorable for protecting against bone loss. Due to low FSH levels osteoclastogenesis is likely to be impaired, and inhibition of osteoblastogenesis through mesenchymal stem cells is decreased. At the same time, significantly increased progesterone and estrogen levels support high bone density. There are numerous direct and indirect actions of estrogen to enhance bone quality.

## ACTH and glucocorticoids

Adrenocorticotropic hormone (ACTH) levels increase due to placental production of ACTH and corticotropin – releasing hormone (CRH) and enhanced pituitary response to CRH [81]. In such conditions, the pituitary becomes less sensitive to the negative feedback of cortisol. These changes also lead to increased cortisol levels, and an increase in cortisol-binding globulin is followed by elevated total cortisol levels. ACTH activates melanocortin 2 receptor (MC2R) on osteoblasts and enhances the upregulation of vascular endothelial growth factor (VEGF) and alpha-2-macroglobulin (A2M), which subsequently stimulates TGF-β synthesis, leading to osteoblast differentiation (82, 83). Supraphysiological glucocorticoid levels suppress osteoblastogenesis and enhance osteoblast apoptosis (84). They promote oxidative stress and reactive oxygen species (ROS) in osteoblasts, impairing osteoblast differentiation (77). In addition, increased levels of cortisol can promote adipogenesis due to a shift in the differentiation of stromal progenitor cells (84). Glucocorticoids restrict the secretion of Wnt and bone morphogenetic proteins (BMP) (77, 85, 86). They also do so in an indirect way by suppressing growth factors (87). Glucocorticoids influence the development and survival of osteoclasts mainly indirectly, via osteoblasts, by modulating RANKL/OPG expression (74). Osteoblastic expression of RANKL is increased, while glucocorticoids decrease OPG secretion by osteoblasts. In line with these actions, glucocorticoids also promote the post-transcriptional upregulation of collagenase-3, which further augments osteoclast-mediated bone resorption (74) (**Figures 2**–**4**). ACTH exerts a positive effect on osteoblasts both through receptors and a network of regulatory proteins. Simultaneously, it increases cortisol levels, resulting in the opposite effect. Cortisol levels in pregnancy are higher than in the non-pregnant state. It is not known which action prevails.

## TSH and thyroid hormones

### TSH

In the first trimester, thyroid-stimulating hormone (TSH) concentrations fall due to human chorionic gonadotropin (hCG) stimulation of the TSH receptor. TSH level rises in the second and third trimesters (88, 89). TSH receptors are present on osteoclasts and osteoblasts, enabling TSH to directly regulate bone function (90). Studies have demonstrated the inhibitory effect of recombinant human TSH on osteoclast formation through upregulating OPG expression and downregulating RANKL expression in osteoblasts (91). In contrast, adding TSH to osteogenic medium enhanced the expression of osteogenic markers and markedly elevated Wnt5a levels in embryonic stem cells (ESCs) (92). Multiple investigations have reported alterations in bone turnover markers (BMTs) within days following rhTSH administration, specifically CTX and NTX levels decreased, whereas a bone formation marker, procollagen type 1 N-terminal propeptide (P1NP), increased (90, 93). TSH also exerts effects on bone indirectly through downregulation of TNF-α, a cytokine that promotes osteoclast differentiation and contributes to bone loss (94, 95). Among other mechanisms, TSH inhibits JNK1/2 and IκBα phosphorylation and c-Jun and p65 nuclear translocation—key steps required for TNF-α production (96). Moreover, TSHR overexpression attenuates AP-1 and NFκB binding activity in response to RANKL and TNF-α/IL-1 stimulation (97).

### Thyroid hormones

In the first trimester hCG-mediated thyroid stimulation supports a slight increase in fT4 (88). Further, a gradual decrease in fT4 follows an estrogen-driven increase in thyroxine-binding globulin (TBG) concentrations, increased plasma volume, and placental deiodinase activity (89). fT3 changes in a similar pattern to fT4. Thyroid receptors (TRs) α1 and β1 are present in the nucleus or in the cytoplasm of bone cells (98). TRs are also expressed on the cell membrane, i.e., monocarboxylate transporter 8 for T4 and 10 for T3 (MCT8, MCT 10) (99). Deiodinases (DIOs) mediate the effect of thyroid hormones on bone cells. DIO2 is present in osteoblasts, and DIO3 in both osteoblasts and osteoclasts (100). Thyroid hormones promote osteoblast differentiation through the BMP/Smad signaling pathway (101). They also regulate osteoblast differentiation by acting on the IGF-1 signaling pathway. Additionally, their metabolites, e.g. 2,5 diiodothyronine and reverse triiodothyronine (T2 and rT3), stimulate IGF-1 mRNA synthesis (102). It was revealed that triiodothyronine (T3) enhances osteocalcin (Ocn) production in osteoblast-like cells *in vitro* (103). T3 elevates the RANKL/OPG ratio in mice. This activation of osteoclasts is mediated through the β2-adrenergic receptor (AR) pathway (104). Human data indicate that thyroid hormones play a role in bone mass maintenance (105). TSH and thyroid hormones exert effects both on osteoblasts and osteoclasts. They promote osteogenesis when their concentrations are normal. However, it should be noted that thyroid diseases are associated with a negative impact on bone health.

## Discussion

Data on the pathophysiology of PLO are very limited. However, it is known that the key regulatory mechanism of maintaining bone quality is the balance between the actions of osteoblasts and osteoclasts.

The RANKL/OPG pathway serves as the primary regulatory axis for bone metabolism (**Figure 3**). If there is an enhanced secretion of RANKL, the proliferation of osteoclasts increases. If OPG secretion prevails, osteoclastogenesis is inhibited, and BMD can improve (106). The well-known hormone to promote osteoclast activity is PRL. Its increase during pregnancy and breastfeeding is thought to be the main cause of PLO. The markedly elevated PRL level is an effect of estrogen’s action on lactotroph cells (33). Another crucial hormone for impaired bone density is PTHrP. PRL contributes to its secretion from the breasts (31, 32).

Moreover, an increase in cortisol level may be a significant contributor to osteoclast formation in pregnancy (74). Its increase in pregnancy is markedly pronounced.

Another important player is a GH, which influences both osteoblasto- and osteoclastogenesis. Maintaining balance between these processes is very important for bone health (48, 53, 107).

Physiological changes in TSH and thyroid hormone concentrations during pregnancy can mimic those observed in hyperthyroidism or hypothyroidism. Although decreased TSH levels may suggest hyperthyroidism, they often decline because human chorionic gonadotropin (hCG) mimics TSH and binds to TSHR. This is not typically associated with pathological thyroid hormone overproduction. Similarly, reduced thyroid hormone levels may mimic hypothyroidism, but this is often due to increased TBG levels rather than impaired hormone production (88, 89, 108). Consequently, these physiological fluctuations in TSH and thyroid hormone concentrations pose an additional challenge in determining the pathogenesis of PLO.

Estrogens secreted in pregnancy seem to be a major protective factor for bone quality (74, 77).

In summary, the pathophysiology of PLO involves a complex hormonal network in which the balance between bone formation and resorption is critical. While estrogens, progesterone, and intermittent PTH act as primary protective factors for BMD, the markedly elevated levels of PRL, cortisol, and PTHrP during pregnancy drive osteoclast activity, potentially tipping the scale toward excessive bone loss. Interactions between hormones during pregnancy are complex. Determining their impact on the possibility of PLO requires both advanced *in vitro* studies and prospective population-based studies among women of reproductive age.
